# Angry facial expressions bias gender categorization in children and adults: behavioral and computational evidence

**DOI:** 10.3389/fpsyg.2015.00346

**Published:** 2015-03-26

**Authors:** Laurie Bayet, Olivier Pascalis, Paul C. Quinn, Kang Lee, Édouard Gentaz, James W. Tanaka

**Affiliations:** ^1^Laboratoire de Psychologie et Neurocognition, University of Grenoble-AlpsGrenoble, France; ^2^Laboratoire de Psychologie et Neurocognition, Centre National de la Recherche ScientifiqueGrenoble, France; ^3^Department of Psychological and Brain Sciences, University of DelawareNewark, DE, USA; ^4^Dr. Eric Jackman Institute of Child Study, University of TorontoToronto, ON, Canada; ^5^Faculty of Psychology and Educational Sciences, University of GenevaGeneva, Switzerland; ^6^Department of Psychology, University of VictoriaVictoria, BC, Canada

**Keywords:** face, emotion, gender, children, representation, stereotype

## Abstract

Angry faces are perceived as more masculine by adults. However, the developmental course and underlying mechanism (bottom-up stimulus driven or top-down belief driven) associated with the angry-male bias remain unclear. Here we report that anger biases face gender categorization toward “male” responding in children as young as 5–6 years. The bias is observed for both own- and other-race faces, and is remarkably unchanged across development (into adulthood) as revealed by signal detection analyses (Experiments 1–2). The developmental course of the angry-male bias, along with its extension to other-race faces, combine to suggest that it is not rooted in extensive experience, e.g., observing males engaging in aggressive acts during the school years. Based on several computational simulations of gender categorization (Experiment 3), we further conclude that (1) the angry-male bias results, at least partially, from a strategy of attending to facial features or their second-order relations when categorizing face gender, and (2) any single choice of computational representation (e.g., Principal Component Analysis) is insufficient to assess resemblances between face categories, as different representations of the very same faces suggest different bases for the angry-male bias. Our findings are thus consistent with stimulus-and stereotyped-belief driven accounts of the angry-male bias. Taken together, the evidence suggests considerable stability in the interaction between some facial dimensions in social categorization that is present prior to the onset of formal schooling.

## Introduction

Models of face perception hypothesize an early separation of variant (gaze, expression, speech) and invariant (identity, gender, and race) dimensions of faces in a stage called structural encoding (Bruce and Young, 1986; Haxby et al., 2000). Structural encoding consists of the abstraction of an expression-independent representation of faces from pictorial encodings or “snapshots.” This results in the extraction of variant and invariant dimensions that are then processed in a hierarchical arrangement where invariant dimensions are of a higher order than the variant ones (Bruce and Young, 1986).

Facial dimensions, however, interact during social perception. Such interactions may have multiple origins, with some but not all requiring a certain amount of experience to develop. First, they may be entirely stimulus-driven or based on the coding of conjunctions of dimensions at the level of single neurons (Morin et al., 2014). Second, the narrowing of one dimension (Kelly et al., 2007) may affect the processing of another. For example, O'Toole et al. (1996) found that Asian and Caucasian observers made more mistakes when categorizing the gender of other-race vs. own-race faces, indicating that experience affects not only the individual recognition of faces (as in the canonical other-race effect, Malpass and Kravitz, 1969), but a larger spectrum of face processing abilities. Third, perceptual inferences based on experience may cause one dimension to cue for another as smiling does for familiarity (Baudouin et al., 2000). Finally, it has been suggested that dimensions interact based on beliefs reflecting stereotypes, i.e., beliefs about the characteristics of other social groups. For example, Caucasian participants stereotypically associate anger with African ethnicity (Hehman et al., 2014). This latter, semantic kind of interaction was predicted by Bruce and Young (1986) who postulated that (1) semantic processes feedback to all stages of face perception, and (2) all invariant dimensions (such as race, gender) are extracted, i.e., “visually-derived,” at this semantic level. More generally, prejudice and stereotyping may profoundly influence even basic social perception (Johnson et al., 2012; Amodio, 2014) and form deep roots in social cognition (Contreras et al., 2012). Data on the development of these processes have reported an onset of some stereotypical beliefs during toddlerhood (Dunham et al., 2013; Cogsdill et al., 2014) and an early onset of the other-race effect in the first year of life (Kelly et al., 2007, 2009).

One observation that has been interpreted as a top-down effect of stereotyping is the perception of angry faces as more masculine (Hess et al., 2004, 2005, 2009; Becker et al., 2007), possibly reflecting gender biases that associate affiliation with femininity and dominance with masculinity (Hess et al., 2007). Alternatively, cues for angry expressions and masculine gender may objectively overlap, biasing human perception at a bottom-up level. Using a forced-choice gender categorization task with signal detection analyses and emotional faces in adults (Experiment 1) and children (Experiment 2), and several computational models of gender categorization (Experiment 3), we aimed to (1) replicate the effect of anger on gender categorization in adults, (2) investigate its development in children, and (3) probe possible bases for the effect by comparing human performance with that of computational models. If the bias is purely driven by top-down beliefs, then computational models would not be sensitive to it. However, if the bias is driven by bottom-up stimulus-based cues, then we expect computational models to be sensitive to such objective cues. To investigate the impact of different facial dimensions on gender-categorization, both own-race and other-race faces were included as stimuli - the latter corresponding to a more difficult task condition (O'Toole et al., 1996).

## Experiment 1: gender categorization by adults

To assess whether emotional facial expressions bias gender categorization, adults categorized the gender of 120 faces depicting unique identities that varied in race (Caucasian, Chinese), gender (male, female), and facial expression (angry, smiling, neutral). We hypothesized that the angry expression would bias gender categorization toward “male,” and that this effect might be different in other-race (i.e., Chinese in the present study) faces that are more difficult to categorize by gender (O'Toole et al., 1996).

### Materials and methods

#### Participants and data preprocessing

Twenty four adult participants (mean age: 20.27 years, range: 17–24 years, 4 men) from a predominantly Caucasian environment participated in the study. All gave informed consent and had normal or corrected-to-normal vision. The experiment was approved by the local ethics committee (“Comité d'éthique des center d'investigation clinique de l'inter-région Rhône-Alpes-Auvergne,” Institutional Review Board). Two participants were excluded due to extremely long reaction times (mean reaction time further than 2 standard deviations from the group mean). Trials with a reaction time below 200 ms or above 2 standard deviations from each participant's mean were excluded, resulting in the exclusion of 4.70% of the data points.

#### Stimuli

**Figure 1:**
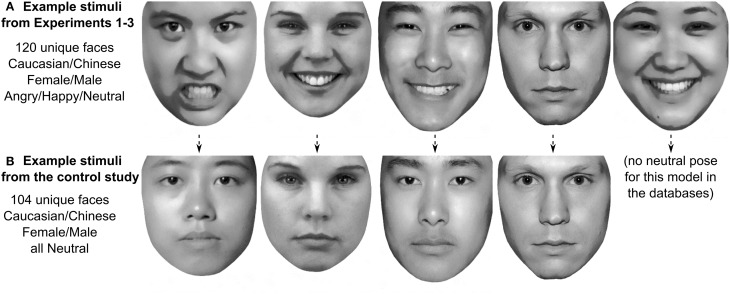
**Example stimuli used in Experiments 1–3 (A) and in the control study (B)**. The identity of the faces used in Experiments 1–3 and in the control study were identical, but in the control study all faces were in neutral expression while faces in Experiments 1–3 had either angry, smiling or neutral expressions. Sixteen of the 120 faces from Experiments 1–3 had no neutral pose in the database.

One hundred twenty face stimuli depicting unique identities were selected from the Karolinska Directed Emotional Face database (Lundqvist et al., 1998; Calvo and Lundqvist, 2008), the NimStim database (Tottenham et al., 2002, 2009), and the Chinese Affective Picture System (Lu et al., 2005) database in their frontal view versions. Faces were of different races (Caucasian, Chinese), genders (female, male), and expressions (angry, neutral, smiling). Faces were gray scaled and placed against a white background; external features were cropped using GIMP. Luminance, contrast, and placement of the eyes were matched using SHINE (Willenbockel et al., 2010) and the Psychomorph software (Tiddeman, 2005, 2011). Emotion intensity and recognition accuracy were matched across races and genders and are summarized in Supplementary Table 1. See Figure 1A for examples of the stimuli used. Selecting 120 emotional faces depicting unique identities for the high validity of their emotional expressions might lead to a potential selection bias, e.g., the female faces that would display anger most reliably might also be the most masculine female faces. To resolve this issue, a control study (Supplementary Material) was conducted in which gender typicality ratings were obtained for the neutral poses of the same 120 faces. See Figure 1B for examples of the stimuli used in the control study.

#### Procedure

Participants were seated 70 cm from the screen. Stimuli were presented using E-Prime 2.0 (Schneider et al., 2002).

A trial began with a 1000–1500 ms fixation cross, followed by a central face subtending a visual angle of about 7 by 7°. Participants completed a forced-choice gender-categorization task. They categorized each face as either male or female using different keys, and which key was associated with which gender response was counterbalanced across participants. The face remained on the screen until the participant responded. Participant response time and accuracy were recorded for each trial.

Each session began with 16 training trials with 8 female and 8 male faces randomly selected from a different set of 26 neutral frontal view faces from the Karolinska Directed Emotional Face database (Lundqvist et al., 1998; Calvo and Lundqvist, 2008). Each training trial concluded with feedback on the participant's accuracy. Participants then performed 6 blocks of 20 experimental trials, identical to training trials without feedback. Half of the blocks included Caucasian faces and half included Chinese faces. Chinese and Caucasian faces were randomly ordered across those blocks. The blocks alternated (either as Caucasian-Chinese-Caucasian… or as Chinese-Caucasian-Chinese…, counterbalanced across participants), with 5 s mandatory rest periods between blocks.

#### Data analysis

Analyses were conducted in Matlab 7.9.0529 and R 2.15.2. Accuracy was analyzed using a binomial Generalized Linear Mixed Model (GLMM) approach (Snijders and Bosker, 1999) provided by R packages lme4 1.0.4 (Bates et al., 2013) and afex 0.7.90 (Singmann, 2013). This approach is robust to missing (excluded) data points and is more suited to binomial data than the Analysis of Variance which assumes normality and homogeneity of the residuals. Accuracy results are presented in the Supplementary Material (Supplementary Figure 1, Supplementary Tables 2, 3). Inverted reaction times from correct trials were analyzed using a Linear Mixed Model (LMM) approach (Laird and Ware, 1982) with the R package nlme 3.1.105 (Pinheiro et al., 2012). Inversion was chosen over logarithm as variance-stabilizing transformation because it led to better homogeneity of the residuals. Mean gender typicality ratings obtained in a control study (Supplementary Material) were included as a covariate in the analysis of both accuracy and reaction times. Finally, signal detection theory parameters (d′, c-bias) were derived from the accuracies of each participant for each condition using the female faces as “signal” (Stanislaw and Todorov, 1999), and then analyzed using repeated measures ANOVAs. Because female faces were used as the “signal” category in the derivation, the conservative bias (c-bias) is equivalent to a male bias. Data and code are available online at http://dx.doi.org/10.6084/m9.figshare.1320891.

### Results

#### Reaction times

**Figure 2:**
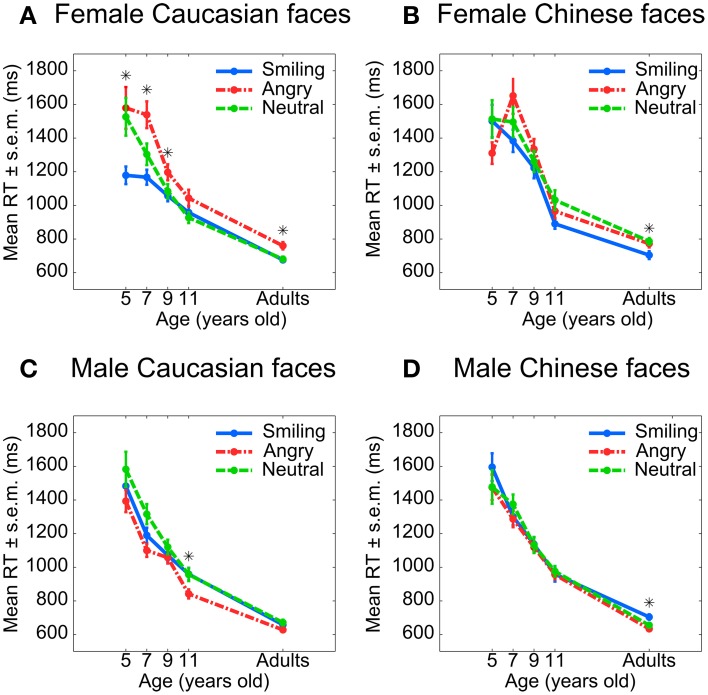
**Reaction times for gender categorization in Experiments 1 (adults) and 2 (children)**. Only reaction times from correct trials are included. Each star represents a significant difference between angry and smiling faces (paired Student *t*-tests, *p* < 0.05, uncorrected). **Top:** Caucasian **(A)** and Chinese **(B)** female faces. **Bottom:** Caucasian **(C)** and Chinese **(D)** male faces.

A Race-by-Gender-by-Emotion three-way interaction was significant in the best LMM of adult inverse reaction times (Table 1). It stemmed from (1) a significant Race-by-Emotion effect on male [χ^2^_(2)_ = 6.48, *p* = 0.039] but not female faces [χ^2^_(2)_ = 4.20, *p* = 0.123], due to an effect of Emotion on Chinese male faces [χ^2^_(2)_ = 8.87, *p* = 0.012] but not Caucasian male faces [χ^2^_(2)_ = 2.49, *p* = 0.288]; and (2) a significant Race-by-Gender effect on neutral [χ^2^_(1)_ = 4.24, *p* = 0.039] but not smiling [χ^2^_(1)_ = 3.31, *p* = 0.069] or angry [χ^2^_(1)_ = 0.14, *p* = 0.706] faces. The former Race-by-Emotion effect on male faces was expected and corresponds to a ceiling effect on the reaction times to Caucasian male faces. The latter Race-by-Gender effect on neutral faces was unexpected and stemmed from an effect of Race in female [χ^2^_(1)_ = 7.91, *p* = 0.005] but not male neutral faces [χ^2^_(1)_ = 0.28, *p* = 0.600] along with the converse effect of Gender on Chinese [χ^2^_(1)_ = 5.16, *p* = 0.023] but not Caucasian neutral faces [χ^2^_(1)_ = 0.03, *p* = 0.872]. Indeed, reaction time for neutral female Chinese faces was relatively long, akin to that for angry female Chinese faces (Figure 2B) and unlike that for neutral female Caucasian faces (Figure 2A). Since there was no hypothesis regarding this effect, it will not be discussed further.

**Table 1:** **Best LMM of adult inverse reaction time from correct trials**.

**Effect**	**d.f**.	**χ^2^**	***p***
(Intercept)	1	334.15	<0.001
Race	1	2.95	0.086
Gender^*^	1	6.17	0.013
Emotion	2	0.07	0.967
Mean gender typicality rating^*^	1	25.97	<0.001
Gender-by-emotion^*^	2	32.13	<0.001
Race-by-emotion^*^	2	6.45	0.040
Race-by-gender	1	0.09	0.761
Race-by-gender-by-emotion^*^	2	7.56	0.023

*The model also included a random intercept and slope for participants. Significant effects are marked by an asterisk*.

Importantly, the interaction of Gender and Emotion in reaction time was significant for both Caucasian [χ^2^_(2)_ = 18.59, *p* < 0.001] and Chinese [χ^2^_(2)_ = 19.58, *p* < 0.001] faces. However, further decomposition revealed that it had different roots in Caucasian and Chinese faces. In Caucasian faces, the interaction stemmed from an effect of Emotion on female [χ^2^_(2)_ = 14.14, *p* = 0.001] but not male faces [χ^2^_(2)_ = 2.49, *p* = 0.288]; in Chinese faces, the opposite was true [female faces: χ^2^_(2)_ = 2.58, *p* = 0.276; male faces: χ^2^_(2)_ = 8.87, *p* = 0.012]. Moreover, in Caucasian faces, Gender only affected reaction time to angry faces [angry: χ^2^_(1)_ = 11.44, *p* = 0.001; smiling: χ^2^_(1)_ = 0.59, *p* = 0.442; neutral: χ^2^_(1)_ = 0.03, *p* = 0.872], whereas in Chinese faces, Gender affected reaction time regardless of Emotion [angry: χ^2^_(1)_ = 25.90, *p* < 0.001; smiling: χ^2^_(1)_ = 7.46, *p* = 0.029; neutral: χ^2^_(1)_ = 5.16, *p* = 0.023].

The impairing effect of an angry expression on female face categorization was clearest on the relatively easy Caucasian faces, while a converse facilitating effect on male face categorization was most evident for the relatively difficult Chinese faces. The effect of Gender was largest for the difficult Chinese faces. The angry expression increased reaction times for Caucasian female faces (Figure 2A) and conversely reduced them for Chinese male faces (Figure 2D).

#### Sensitivity and male bias

A repeated measures ANOVA showed a significant Race-by-Emotion effect on both d′ (Table 2) and male-bias (Table 3).

**Table 2:** **ANOVA of d-prime for adult gender categorization**.

**Fixed effects**	**SS**	**d.f**.	**MS**	***F***	***p***	**η^2^**
Race^*^	17.77	1	17.77	106.38	<0.001	0.38
Emotion^*^	5.91	2	2.96	22.24	<0.001	0.13
Race-by-emotion^*^	3.56	2	1.78	13.84	<0.001	0.08
Error	5.40	42				
Total	47.30	131				

*The ANOVA also included a random factor for the participants, along with its interactions with both Race and Emotion. Significant effects are marked by an asterisk*.

**Table 3:** **ANOVA of male-bias for adult gender categorization**.

**Fixed effects**	**SS**	**d.f**.	**MS**	***F***	***p***	**η^2^**
Race^*^	17.16	1	17.16	93.03	<0.001	0.35
Emotion^*^	8.24	2	4.12	40.57	<0.001	0.17
Race-by-emotion^*^	3.18	2	1.59	12.71	<0.001	0.06
Error	5.26	42	0.13			
Total	49.55	131				

*The ANOVA also included a random factor for the participants, along with its interactions with both Race and Emotion. Significant effects are marked by an asterisk*.

**Figure 3:**
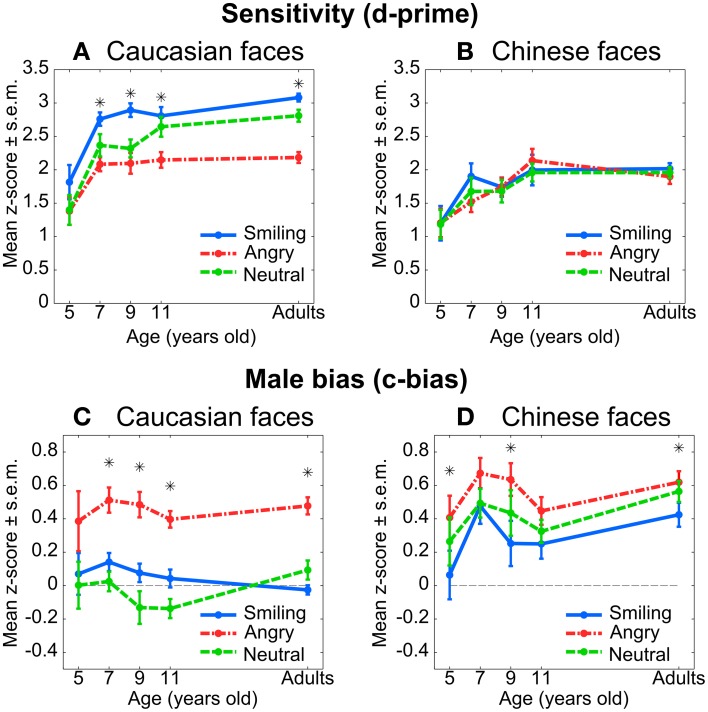
**Sensitivity and male bias for gender categorization in Experiments 1 (adults) and 2 (children)**. Female faces were used as “signal” class. Each star represents a significant difference between angry and smiling faces (paired Student *t*-tests, *p* < 0.05, uncorrected). **Top:** Sensitivity for Caucasian **(A)** and Chinese **(B)** faces. **Bottom:** Male bias for Caucasian **(C)** and Chinese **(D)** faces.

Sensitivity was greatly reduced in Chinese faces (η^2^ = 0.38, i.e., a large effect), replicating the other-race effect for gender categorization (O'Toole et al., 1996). Angry expressions reduced sensitivity in Caucasian but not Chinese faces (Figures 3A,B). Male bias was high overall, also replicating the finding by O'Toole et al. (1996). Here, in addition, we found that (1) the male bias was significantly enhanced for Chinese faces (η^2^ = 0.35, another large effect), and (2) angry expressions also enhanced the male bias, as predicted, in Caucasian and Chinese faces (η^2^ = 0.17, a moderate effect)—although to a lesser extent in the latter (Figures 3C,D). Since Emotion affects the male bias but not sensitivity in Chinese faces, it follows that the effect of Emotion on the male bias is not solely mediated by its effect on sensitivity.

Further inspection of the experimental effect on the hit rate (female trials) and false alarm rate (male trials) confirmed, however, that the overall performance was at ceiling on male faces, as repeated measures ANOVAs revealed a significant interactive effect of Race and Emotion on the hit rate [*F*_(2, 42)_ = 12.71, *p* < 0.001, η^2^ = 0.07] but no significant effect of Race, Emotion, or their interaction on the false alarm rate (all *p*s > 0.05). In other words, the effects of Race and Emotion on d′ and male bias were solely driven by performance on female faces. Accuracy results are presented in the Supplementary Material (Supplementary Figure 1, Supplementary Table 2).

### Discussion

The effect of anger on gender categorization was evident on reaction time, as participants were (1) slower when categorizing the gender of angry Caucasian female faces, (2) slower with angry Chinese female faces, and (3) quicker with angry Chinese male faces. Interestingly, the angry expression reduced sensitivity (d′) of gender categorization in own-race (Caucasian), but not in other-race (Chinese) faces. In other words, angry expressions had two dissociable effects on gender categorization: (1) they increased difficulty when categorizing own-race faces, and (2) they increased the overall bias to respond “male.”

The results are consistent with the hypothesis of a biasing effect of anger that increases the tendency to categorize faces as male. However, a ceiling effect on accuracy for male faces made it impossible to definitively support this idea. To firmly conclude in favor of a true bias, it should be observed that angry expressions both hinder female face categorization (as was observed) and enhance male face categorization (which was not observed). While a small but significant increase in accuracy for angry vs. happy Chinese male faces was observed (Supplementary Figure 1D), there was no significant effect on the false alarm rate (i.e., accuracy on male trials).

Different from the present results, O'Toole et al. (1996) did not report an enhanced male bias for other-race faces (Japanese or Caucasian) faces, although they did find an effect on d′ that was replicated here, along with an overall male bias. The source of the difference is uncertain, one possibility being that the greater difficulty of the task used in O'Toole et al. (a 75 ms presentation of each face followed by a mask) caused a male bias for own-race faces, or that the enhanced male bias to other-race faces found in the present study does not generalize to all types of other-race faces. Finally, O'Toole et al. (1996) found that female participants had displayed higher accuracy on a gender categorization task than male participants. However, the sample for the current study did not include enough male participants to allow us to analyze this possible effect.

## Experiment 2: gender categorization in children

One way to understand the male bias is to investigate its development. There is a general consensus that during development we are ”becoming face experts” (Carey, 1992) and the immature face processing system that is present at birth will develop with experience until early adolescence (Lee et al., 2013). If the angry male bias develops through extensive experience with peers observing male aggression during the school years, it follows that the angry male bias should be smaller in children than in adults and that the bias would increase during the school years, a time period when children observe classmates (mostly males) engaging in aggressive acts inclusive of fighting and bullying.

In Experiment 2, we conducted the same gender categorization task as in Experiment 1 with 64 children aged from 5 to 12. The inclusion of children in the age range from 5 to 6, as well the testing of 7–8, 9–10, and 11–12 year-olds, is important from a developmental perspective. Experiment 2 should additionally allow us to (1) overcome the ceiling effect on gender categorization for male faces that was observed in Experiment 1 (as children typically perform worse than adults in gender categorization tasks, e.g., Wild et al., 2000), and (2) determine the developmental trajectory of the biasing effect of anger in relation to increased experience with processing own-race (Caucasian) but not other-race (Chinese) faces. While facial expression perception also develops over childhood and even adolescence (Herba and Phillips, 2004), recognition performance for own-race expressions of happiness and anger have been reported to be at ceiling from 5 years of age (Gao and Maurer, 2010; Rodger et al., 2015).

### Methods

#### Participants and preprocessing

Thirteen 5–6 year-olds (9 boys), 16 7–8 year-olds (3 boys), 15 9–10 year-olds (9 boys), and 14 11–12 year-olds (3 boys) from a predominantly Caucasian environment were included in the final sample. These age groups were chosen a priori due to the minimal need to re-design the experiment: children from 5 to 6 years of age may complete computer tasks and follow directions. A range of age groups was then selected from 5 to 6 years old onwards, covering the developmental period from middle to late childhood, and the time when children begin formal schooling. The experiment was approved by the University of Victoria Human Research Ethics Board and informed parental consent was obtained. Six additional participants were excluded due to non-compliance (*n* = 1) or very slow reaction times for their age (*n* = 5). Additionally, trials from participants were excluded if their reaction times were extremely short (less than 600, 500, 400, or 300 ms for 5–6 year olds, 7–8 year olds, 9–10 year olds, or 11–12 year olds, respectively) or further than 2 standard deviations away from the participant's own distribution. Such invalid trials were handled as missing values, leading to the exclusion of 11.35% data points in the 5–6 years olds, 5.57% in the 7–8 year olds, 5.28% in the 9–10 year olds, and 4.88% in the 11–12 year olds. The cut-offs used to exclude trials with very short reaction times were selected graphically based on the distribution of reaction times within each age group.

#### Stimuli, procedure, and data analysis

Stimuli, task, procedure, and data analysis methods were identical to that of Experiment 1 except for the following: Participants were seated 50 cm from the screen so that the faces subtended a visual angle of approximately 11 by 11°. Due to an imbalance in the gender ratio across age groups, the participant's gender was included as a between-subject factor in the analyses. Data and code are available online at http://dx.doi.org/10.6084/m9.figshare.1320891.

### Results

#### Reaction times

There was a significant Race-by-Gender-by-Emotion interaction in the best linear mixed model (LMM) of children's inverse reaction times from correct trials (Table 4), along with a three-way Age-by-Gender-by-Participant gender interaction, an Age-by-Race-by-Emotion interaction, and a Participant gender-by-Gender-by-Emotion interaction.

**Table 4:** **Best LMM of children's inverted reaction times from correct trials**.

**Fixed effects**	**d.f**.	**χ^2^**	***p***
(Intercept)	1	113.97	<0.001
Race^*^	1	14.07	<0.001
Gender^*^	1	4.00	0.046
Emotion^*^	2	7.27	0.026
Age^*^	3	11.18	0.011
Participant gender	1	0.16	0.687
Mean gender typicality rating^*^	1	75.34	<0.001
Race-by-gender	1	0.38	0.539
Gender-by-emotion^*^	2	13.32	0.001
Race-by-emotion^*^	2	12.97	0.002
Age-by-race^*^	3	12.17	0.007
Age-by-gender^*^	3	8.80	0.032
Age-by-emotion	6	8.58	0.198
Participant gender-by-gender	1	0.50	0.480
Participant gender-by-emotion	2	3.45	0.179
Participant gender-by-age	3	3.21	0.360
Race-by-gender-by-emotion^*^	2	9.89	0.007
Age-by-race-by-emotion^*^	6	18.66	0.005
Age-by-gender-by-participant gender^*^	3	9.35	0.025
Participant gender-by-gender-by-emotion^*^	2	8.16	0.017

*The model also included a random intercept and slope for the participants. Significant effects are marked by an asterisk*.

The interaction of Age, Gender, and Participant gender was due to a significant Gender-by-Participant gender interaction in the 11–12 year olds [χ^2^_(1)_ = 6.19, *p* = 0.013], with no significant sub-effects (*p*s > 0.05). The interaction of Gender, Emotion, and Participant gender was due to the effect of Gender on angry faces reaching significance in female (female faces, inverted RT: 9.35 ± 3.67.10^−4^ ms^−1^; male faces: 10.67 ± 3.51.10^−4^ ms^−1^) but not male participants (female faces, inverted RT: 8.88 ± 3.24.10^−4^ ms^−1^; male faces: 9.72 ± 3.26.10^−4^ ms^−1^), although the effect had the same direction in both populations. Importantly, however, the overall Gender-by-Emotion interaction was significant in both male [χ^2^_(2)_ = 7.44, *p* = 0.024] and female participants [χ^2^_(2)_ = 52.41, *p* < 0.001]. The interaction of Race and Emotion with Age reflected the shorter reaction times of 5–6 year olds when categorizing the gender of Caucasian vs. Chinese smiling faces [χ^2^_(2)_ = 7.40, *p* = 0.007], also evidenced by a significant Race-by-Age interaction for smiling faces only [χ^2^_(3)_ = 10.11, *p* = 0.018]. Faster responses to smiling Caucasian faces by the youngest participants probably reflect the familiarity, or perception of familiarity in these stimuli.

Finally, the interactive effect of Gender and Emotion on reaction times was significant in Caucasian [χ^2^_(2)_ = 49.81, *p* < 0.001] but not Chinese faces [χ^2^_(2)_ = 2.25, *p* = 0.325] leading to a Race-by-Gender-by-Emotion interaction. Further decomposition confirmed this finding: Race significantly affected reaction times for male [χ^2^_(1)_ = 19.52, *p* < 0.001] but not female angry faces [χ^2^_(1)_ = 1.86, *p* = 0.173], Gender affected reaction times for Caucasian [χ^2^_(1)_ = 17.01, *p* < 0.001] but not Chinese angry faces [χ^2^_(1)_ = 0.48, *p* = 0.489], and Emotion significantly affected the reaction times for Caucasian female [χ^2^_(2)_ = 29.88, *p* < 0.001] but not Chinese female [χ^2^_(2)_ = 3.82, *p* = 0.148] or male faces [χ^2^_(2)_ = 5.13, *p* = 0.077].

Children were slower when categorizing the gender of angry vs. happy Caucasian female faces (Figure 2A), and slightly faster when categorizing the gender of angry vs. happy Caucasian male faces (Figure 2C). The interaction of Gender and Emotion was present in all participants but most evident in female participants. It was absent in Chinese faces. In other words, an angry expression slows gender categorization in own-race (Caucasian) but not in other-race (Chinese) faces.

#### Sensitivity and male bias

ANOVAs with participant as a random factor showed a small, but significant Race-by-Emotion interaction on sensitivity (d′, Table 5, η^2^ = 0.02) and male-bias (c-bias, Table 6, η^2^ = 0.03). Neither for sensitivity nor for male-bias did the Race-by-Emotion interaction or its subcomponents interact with Age.

**Table 5:** **ANOVA of d′ for children's gender categorization**.

**Fixed effects**	**SS**	**d.f**.	**MS**	***F***	***p***	**η^2^**
Race^*^	28.32	1	28.32	80.59	<0.001	0.13
Emotion^*^	6.14	2	3.07	12.65	<0.001	0.03
Age^*^	21.04	3	7.01	6.40	0.001	0.09
Participant gender	4.15	1	4.15	3.79	0.057	0.02
Race-by-emotion^*^	4.55	2	2.27	8.58	<0.001	0.02
Age-by-race	2.56	3	0.85	2.42	0.076	0.01
Age-by-emotion	0.89	6	0.15	0.61	0.719	<0.01
Age-by-gender-by-emotion	1.12	6	0.19	0.71	0.644	0.01
Participant gender-by-race	0.83	1	0.83	2.35	0.131	<0.01
Participant gender-by-emotion^*^	3.99	2	1.99	8.21	0.001	0.02
Participant gender-by-gender-by-emotion	0.36	2	0.18	0.68	0.511	<0.01
Age-by-participant gender	3.63	3	1.21	1.10	0.356	0.02
Error	28.07	106	0.27			
Total	223.56	347				

*The ANOVA also included a random factor for the participants along with its interactions with both Race and Emotion. Significant effects are marked by an asterisk*.

**Table 6:** **ANOVA of male-bias for children's gender categorization**.

**Fixed effects**	**SS**	**d.f**.	**MS**	***F***	***p***	**η^2^**
Race^*^	4.88	1	4.88	53.50	<0.001	0.07
Emotion^*^	7.65	2	3.83	36.49	<0.001	0.12
Age	0.50	3	0.17	0.34	0.797	0.01
Participant gender	0.49	1	0.49	0.99	0.324	0.01
Race-by-emotion^*^	1.88	2	0.94	17.08	<0.001	0.03
Age-by-race	0.68	3	0.23	2.5	0.070	0.01
Age-by-emotion	0.44	6	0.07	0.7	0.654	0.01
Age-by-gender-by-emotion	0.12	6	0.02	0.35	0.909	<0.01
Participant gender-by-race	0.03	1	0.03	0.31	0.578	<0.01
Participant gender-by-emotion	0.26	2	0.13	1.25	0.290	<0.01
Participant gender-by-gender-by-emotion	0.27	2	0.13	2.42	0.093	<0.01
Age-by-participant gender	0.63	3	0.21	0.43	0.734	0.01
Error	5.80	106	0.06			
Total	66.35	347				

*The ANOVA also included a random factor for participant, along with its interactions with both Race and Emotion. Significant effects are marked by an asterisk*.

Two additional effects on sensitivity (d′) can be noted (Table 5). First, there was a significant effect of Age as sensitivity increased with age (η^2^ = 0.09). Second, there was an interactive effect of Emotion and Participant gender that stemmed from female participants having higher sensitivity than male participants on happy [*F*_(1, 114)_ = 9.14, *p* = 0.003] and neutral [*F*_(1, 114)_ = 18.39, *p* < 0.001] but not angry faces [*F*_(1, 114)_ = 0.39, *p* = 0.533]. Emotion affected the overall sensitivity of both female [*F*_(1, 102)_ = 21.07, *p* < 0.001] and male participants [*F*_(1, 72)_ = 4.69, *p* = 0.014].

The pattern of the interactive effect for Race and Emotion was identical to that found in adults: anger reduced children's sensitivity (d′) to gender in Caucasian faces (Figure 3A), but not in the already difficult Chinese faces (Figure 3B). This pattern is remarkably similar to that found in reaction times. In contrast, anger increased the male-bias in Caucasian (Figure 3C) as well as Chinese faces (Figure 3D), although to a lesser extent in the latter category. In other words, the biasing effect of anger cannot be reduced to an effect of perceptual difficulty. Further analyses revealed that Race and Emotion affected the hit (female trials) and false alarm (male trials) rates equally, both as main and interactive effects [Race-by-Emotion effect on hit rate: *F*_(2, 106)_ = 10.70, *p* < 0.001, η^2^ = 0.02; on false alarm rate: *F*_(2, 114)_ = 13.48, *p* < 0.001, η^2^ = 0.03]. That is, the male-biasing effect of anger is evident by its interfering effect during female trials as well as by its converse facilitating effect during male trials. Accuracy results are presented in the Supplementary Material (Supplementary Figure 1, Supplementary Table 3).

These last observations are compatible with the idea that angry expressions bias gender categorization. The effect can be observed across all ages and even with unfamiliar Chinese faces, although in a diminished form. The biasing effect of anger toward “male” does not seem to depend solely on experience with a particular type of face and is already present at 5–6 years of age.

### Discussion

The results are consistent with a male-biasing effect of anger that is in evidence as early as 5–6 years of age and that is present, but less pronounced in other-race (Chinese) than in own-race (Caucasian) faces. The ceiling effect observed in Experiment 1 on the gender categorization of male faces (i.e., the false alarm rate) was sufficiently overcome so that the male-biasing effect of anger could be observed in male as well as female trials.

Participant gender interacted with Emotion on sensitivity and with Emotion and Gender on the reaction times of children. This finding partly replicates the finding by O'Toole et al. (1996) that female participants present higher face gender categorization sensitivity (d′) than male participants, particularly with female faces. Here, we further showed that in children, this effect is limited to neutral and happy faces, and does not generalize to angry faces.

It is perhaps surprising that anger was found to affect the male-bias on Chinese as well as Caucasian faces, but only affected sensitivity (d′) and reaction times on Caucasian faces. Two dissociable and non-exclusive effects of angry expressions may explain this result. First, angry expressions may be less frequent (e.g., Malatesta and Haviland, 1982), which would generally slow down and complicate gender categorization decisions for familiar (Caucasian) but not for the already unfamiliar (Chinese) faces. This effect is not a bias and should only affect sensitivity and reaction time. Second, angry expressions may bias gender categorization toward the male response by either lowering the decision criterion for this response (e.g., as proposed by Miller et al., 2010) or adding evidence for it. It naturally follows that such an effect should be evident on the male-bias (c-bias), but not on sensitivity. Should it be evident in reaction time, as we initially predicted? Even if a bias does not affect the overall rate of evidence accumulation, it should provide a small advantage on reaction time for “male” decisions, and conversely result in a small delay on reaction time for “female” decisions. While this effect would theoretically not depend on whether the face is relatively easy (own-race) or difficult (other-race) to categorize, it is possible that it would be smaller in other-race faces for two reasons: (1) the extraction of the angry expression itself might be less efficient in other-race faces, leading to a smaller male-bias; and (2) the small delaying or quickening effect of anger could be masked in the noisy and sluggish process of evidence accumulation for other-race faces.

Three possible mechanisms could explain the male-biasing effect of angry expressions: Angry faces could be categorized as “male” from the resemblance of cues for angry expressions and masculine gender, from experience-based (Bayesian-like) perceptual inferences, or from belief-based inferences (i.e., stereotype). Of interest is that the male-biasing effect of anger was fairly constant from 5 to 12 years of age. There are at least two reasons why the male-biasing effect of anger would already be present in adult form in 5–6 years olds: (1) the effect could develop even earlier than 5–6 years of age, or (2) be relatively independent of experience (age, race) and maturation (age). Unfortunately, our developmental findings neither refute nor confirm any of the potential mechanisms for a male-bias. Indeed, any kind of learning—whether belief-based or experience-based - may happen before the age of 5 years without further learning afterwards. For example, Dunham et al. (2013) evidenced racial stereotyping in children as young as 3 years of age using a race categorization task with ambiguous stimuli. Similar findings were reported on social judgments of character based on facial features (Cogsdill et al., 2014). Conversely, the resemblance of cues between male and angry faces would not necessarily predict a constant male-biasing effect of anger across all age groups: for example, the strategy used for categorizing faces based on gender may well vary with age so that the linking of cues happens at one age more than another because children use one type of cue more than another at some ages. For example, it has been established that compared to adults, children rely less on second-order relations between features for various face processing tasks, and more on individual features, external features, or irrelevant paraphernalia, with processing of external contour developing more quickly than processing of feature information (Mondloch et al., 2002, 2003). Holistic processing, however, appears adult-like from 6 years of age onwards (Carey and Diamond, 1994; Tanaka et al., 1998; Maurer et al., 2002). Therefore, each age group presents a unique set, or profile, of face processing strategies that may be more or less affected by the potential intersection of cues between male and angry faces. Whichever mechanism or mechanisms come to be embraced on the basis of subsequent investigations, what our developmental findings do indicate is that the angry-male bias is not dependent on peers observing an association between males and aggression during the school age years.

## Experiment 3: computational models of gender categorization

To determine if the effect of anger on gender categorization could be stimulus driven, i.e., due to the resemblance of cues for angry expressions and masculine gender, machine learning algorithms were trained to categorize the gender of the faces used as stimuli in Experiments 1–2. If algorithms tend to categorize angry faces as being male, as humans do, then cues for anger and masculinity are conjoined in the faces themselves and there should be no need to invoke experience- or belief-based inferences to explain the human pattern of errors.

### Methods

#### Stimuli

Stimuli were identical to those used in Experiments 1, 2.

#### Different computational models

**Figure 4:**
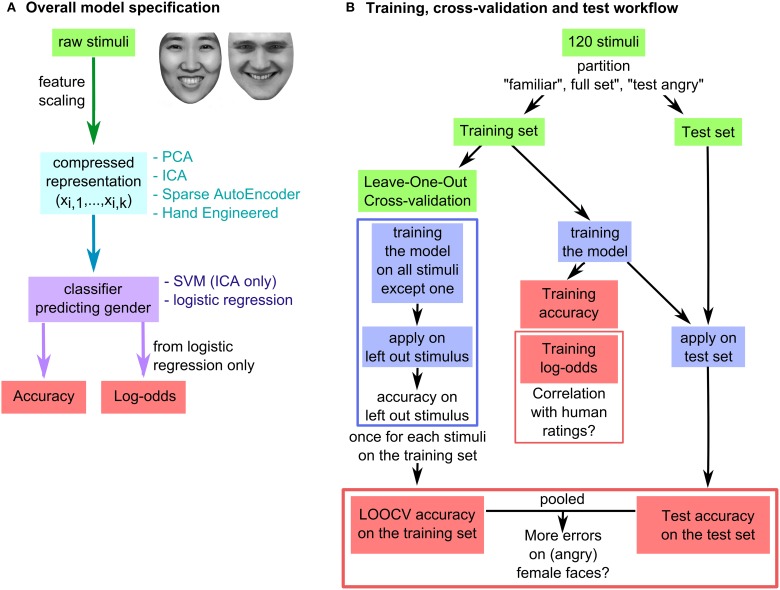
**Computational models. (A)** Overall model specification. Each model had an unsupervised learning step (either PCA, ICA) followed by a supervised learning step (logistic regression or SVM). **(B)** Training, cross validation and test workflow. Stimuli were partitioned into a training set and a test set. Variables used in further analysis were the Leave-One-Out Cross-validation (LOOCV) accuracy, the test accuracy, and the log-odds at training. Human ratings were obtained in the control study (Supplementary Material).

Analyses were run in Matlab 7.9.0529. The raw stimuli were used to train different classifiers (Figure 4A). The stimuli were divided into a training set and a test set that were used separately to obtain different measures of gender categorization accuracy (Figure 4B). Several models and set partitions were implemented to explore different types of training and representations (Table 7; Figure 4A).

**Table 7:** **Representations, classifiers, and face sets used in the computational models of gender categorization**.

**Representation**	**Classifier**		**Training and test faces**			**Sets size**	
			**Partition**	**Training set**	**Test set**	**Training**	**Test**
Principal component analysis (PCA)	Logistic regression	A	“Familiar”	Neutral and happy Caucasian	Angry and Chinese	*n* = 40	*n* = 80
		B	“Full set”	All faces	–	*n* = 120	*n* = 0
		C	“Test angry”	Neutral and happy	Angry	*n* = 80	*n* = 40
Independent component analysis (ICA)	Support vector machine (SVM)	D	“Familiar”	Neutral and happy Caucasian	Angry and Chinese	*n* = 40	*n* = 80
		E	“Full set”	All faces	–	*n* = 120	*n* = 0
		F	“Test angry”	Neutral and happy	Angry	*n* = 80	*n* = 40
Sparse auto-encoder (SAE)	Logistic regression	G	“Familiar”	Neutral and happy Caucasian	Angry and Chinese	*n* = 40	*n* = 80
		H	“Full set”	All faces	–	*n* = 120	*n* = 0
		I	“Test angry”	Neutral and happy	Angry	*n* = 80	*n* = 40
Hand-engineered features (HE)	Logistic regression	J	“Familiar”	Neutral and happy Caucasian	Angry and Chinese	*n* = 40	*n* = 80
		K	“Full set”	All faces	–	*n* = 120	*n* = 0
		L	“Test angry”	Neutral and happy	Angry	*n* = 80	*n* = 40

Different types of representations (Principal Component Analysis, Independent Components Analysis, Sparse Auto-encoder, and Hand-Engineered features; Table 7; Figure 4A) were used because each of them might make different kinds of information more accessible to the classifier; i.e., the cue-dimension relationship that drives human errors may be more easily accessible in one representation than another. Sparse auto-encoded representations are considered the most “objective” of these representations in contrast to other unsupervised representations (Principal Component Analysis, Independent Components Analysis) that use a specific, deterministic method for information compression. Conversely, hand engineered features are the most “human informed” representation, since they were defined in Burton et al. (1993) using human knowledge about what facial features are (eyes, brows, mouth) and about the assumed importance of these features for gender categorization and face recognition. The choice of Principal Component Analysis as an unsupervised representation method (used in models A–C, and as a preprocessing step in models D–F) was motivated by the knowledge that PCA relates reliably to human ratings and performance (O'Toole et al., 1994, 1998) and has been proposed as a statistical analog of the human representation of faces (Calder and Young, 2005).

All models included feature scaling of raw pixels as a first preprocessing step. Models based on Principal Component Analysis (PCA, models A–C) used the first 16 principal components for prediction (75% of variance retained). Models based on Independent Components Analysis (ICA, models D–F) used the Fast-ICA implementation for Matlab (Gävert et al., 2005) that includes PCA and whitening as a preprocessing step. Sparse representations (models G–I) were obtained using the sparse auto-encoder neural network implemented in the NNSAE Matlab toolbox (Lemme et al., 2012). A sparse auto-encoder is a particular kind of neural network that aims to obtain a compressed representation of its input by trial and error. The hand-engineered features used in models J-L were the 11 full-face 2D-features and second-order relations identified in Burton et al. (1993) as conveying gender information (for example, eyebrow thickness, eyebrow to eye distance, etc.).

Most models used a logistic regression classifier because this method provides log-odds that were useful for human validation. Models D–F used the Support Vector Machine Classifier implementation from the SVM-KM toolbox for Matlab (Gaussian kernel, *h* = 1000, quadratic penalization; Canu et al., 2005) because in those models the problem was linearly separable (meaning that using logistic regression was inappropriate and would lead to poor performance).

Each model was trained on a set of faces (the training set, leading to the computation of training set accuracy), and then tested on a different set of faces (the test set, resulting in computation of test accuracy). Accuracy on the training sets was further evaluated using Leave-One-Out cross-validation (LOOCV), which is thought to reflect generalization performance more accurately than training accuracy. Accuracies at test and cross-validation (LOOCV) were pooled together for comparing the performance on (angry) female vs. male faces. See Figure 4B for a schematic representation of this set up.

The partitioning of faces as training and test sets differed across the models (Figure 4B). The partitioning of models A, D, G, and J (“familiar”) was designed to emulate the actual visual experience of human participants in Experiments 1–2. The partitioning for models B, E, H, and K (“full set”) was designed to emphasize all resemblances and differences between faces equally without preconception. The partitioning for models C, F, I, and L (“test angry”) was designed to maximize the classification difficulty of angry faces, enhancing the chance to observe an effect.

#### Human validation

Gender typicality ratings from a control experiment (Supplementary Material) were used to determine how each model accurately captured the human perception of gender: the classifier should find the most gender-typical faces easiest to classify, and vice-versa. Ratings from male and female faces from the training sets were z-scored separately, and the Pearson's correlation between those z-scored ratings and the linear log-odds output from each model at training were computed. The log-odds represent the amount of evidence that the model linearly accumulated in favor of the female response (positive log-odds) or in favor of the male response (negative log-odds). The absolute value of the log-odds was used instead of raw log-odds so that the sign of the expected correlation with gender typicality was positive for both male and female faces and one single correlation coefficient could be computed for male and female faces together. Indeed, the faces with larger absolute log-odds are those that the model could classify with more certainty as male or female: if the model adequately emulated human perception, such faces should also be found more gender typical by humans.

Data and code are available online at http://dx.doi.org/10.6084/m9.figshare.1320891.

### Results

Results are summarized in Table 8 below.

**Table 8:** **Accuracy, correlation with human ratings, and replication of experimental effects by different computational models of gender categorization**.

		**Accuracy (%)**			**Correlation with ratings**		**Female vs. male: Angry faces**			**Female vs. male: All faces**		
		**Training**	**CV**	**Test**	***r***	***p***	**Δ%**	***p***	**χ^2^_(1)_**	**Δ%**	***p***	**χ^2^_(1)_**
PCA	A	82.50	72.50	68.75	0.46	0.003	45.00	0.001	10.16	30.00	<0.001	12.9
	B	92.50	76.67	–	0.23	0.019	35.00	0.013	6.14	6.67	0.388	0.75
	C	81.25	66.25	77.50	0.11	0.357	15.00	0.256	1.29	6.67	0.426	0.64
ICA	D	100.00	85.00	68.75	–	–	50.00	<0.001	10.99	35.00	<0.001	19.18
	E	100.00	85.00	–	–	–	15.00	0.256	1.29	3.33	0.609	0.26
	F	100.00	85.00	72.50	–	–	25.00	0.077	3.14	5.00	0.487	0.48
SAE	G	72.50	50.00	48.75	0.14	0.379	10.00	0.519	0.42	−18.33	0.045	4.03
	H	62.50	50.00	–	−0.05	0.587	−10.00	0.527	0.40	−6.67	0.465	0.53
	I	61.25	53.75	50.00	0.06	0.643	0.00	1.000	0.00	−1.67	0.855	0.03
HE	J	85.00	72.50	62.50	0.11	0.494	−45.00	0.004	8.29	−1.67	0.847	0.04
	K	81.67	76.67	–	0.25	0.012	−40.00	0.006	7.62	−3.33	0.666	0.19
	L	83.75	76.25	62.50	0.24	0.043	−75.00	<0.001	24.00	−30.00	<0.001	13.30

*Models used either Principal Component Analysis (PCA, models A–C), Independent Component Analysis (ICA, models D–F), features generated by a sparse auto-encoder (SAE, models G–I), or hand-engineered features (HE, models J–L). Correlations with ratings are Pearson correlation coefficients between absolute log-odds at training and z-scored gender typicality ratings from humans. Results from the sparse auto-encoder vary at each implementation as the procedure is not entirely deterministic; a single implementation is reported here*.

#### Overall classification performance

Sparse-based models (Table 8, SAE, G–I) performed poorly (around 50% at test and cross-validation) and showed no correlation with human ratings, probably due to the difficulty of training this kind of network on relatively small training sets. Those models were therefore discarded from further discussion. PCA-based models (Table 8, PCA, A–C) on the other hand had satisfactory test (68.75–77.50%) and cross-validation (66.25–76.67%) accuracies, comparable to that of 5–6 year old children (Supplementary Figure 1). ICA- and SVM- based models (Table 8, ICA, D–F) performed, as expected, slightly better than models A-C at training (100%) and cross-validation (85%). However, performance at test (68.75–72.50%) was not better. Models based on hand-engineered features (Table 8, HE, J–L) had test and cross-validation performance in comparable ranges (62.50–76.67%), and their training accuracy (81.00–85.00%) was comparable to that of 85.5% reported by Burton et al. (1993) on a larger set of neutral Caucasian faces (*n* = 179). Most notably, the latter models all included eyebrow width and eye-to-eyebrow distance as significant predictors of gender.

#### Human validation

Classification evidence (absolute log-odds) correlated with z-scored human ratings in 2 of the 3 models from the PCA based model family (Table 8, A,B) as well as in 2 of the 3 models based on hand-engineered features (Table 8, K,L). The highest correlation (Pearson *r* = 0.46, *p* = 0.003) was achieved in model A that used PCA and a training set designed to emulate the content of the participants' visual experience (“familiar”). PCA-based representations might dominate when rating the gender typicality of familiar faces, while a mixture of “implicit” PCA-based and “explicit” feature-based representations might be used when rating the gender typicality of unfamiliar faces.

#### Replication of human errors

Only one of the models (Table 8, D) exhibited an other-race effect, and this effect was only marginal [Δ = −15.00%, *p* = 0.061, χ^2^_(1)_ = 3.52]. Two models actually exhibited a reverse other-race effect, with better classification accuracy on Chinese than Caucasian faces [model C: Δ = 16.67%, *p* = 0.046, χ^2^_(1)_ = 3.97; model K: Δ = 16.67%, *p* = 0.031, χ^2^_(1)_ = 4.66]. Overall, the computational models failed to replicate the other-race effect for human gender categorization that was reported in Experiments 1–2 and in O'Toole et al. (1996).

The pattern of errors from PCA- or ICA-based models (Table 8, A–F) and feature-based models (Table 8, J–L) on female vs. male faces were in opposite directions. Four out of 6 PCA- and ICA- based models made significantly (Table 8, A,B,D) or marginally more mistakes (F) on male vs. female angry faces. Conversely, all 3 feature-based models (Table 8, J–L) made more mistakes on female vs. male angry faces, as did humans in Experiments 1–2. Similar patterns were found when comparing classification performance on all female vs. male faces, although the effect only reached significance in 2 out of 6 PCA- or ICA-based models (Table 8, A,D) and in 1 out of 3 feature-based models (Table 8, L). Hence, two different types of representations led to completely different predictions of human performance, only one of which replicated the actual data. Thus, the features of angry faces resemble that of male faces, potentially biasing gender categorization. However, this information is absent in PCA and ICA representations that actually convey the reverse bias.

Absolute log-odds obtained by the feature-based model J on familiar (neutral and happy Caucasian) faces significantly correlated with mean human (children and adults) accuracy on these faces in Experiments 1–2 (Spearman *r* = 0.39, *p* = 0.013), while the absolute log-odds obtained by the PCA-based model A on those same faces correlated only marginally with human accuracy (Spearman's *r* = 0.28, *p* = 0.077). In other words, feature-based models also better replicated the human pattern of errors in categorizing the gender of familiar faces. See Supplementary Table 4 for a complete report of correlations with human accuracies for models A–C and J–L.

### Discussion

Overall, the results support the idea that humans categorize the gender of faces based on facial features (and second-order relations) more than on a holistic, template-based representation captured by Principal Component Analysis (PCA). In contrast, human ratings of gender typicality tracked feature-based as well as PCA-based representations. This feature-based strategy for gender categorization leads to a confusion between the dimensions of gender and facial expression, at least when the faces are presented statically and in the absence of cues such as hairstyle, clothing, etc. In particular, angry faces tend to be mistaken for male faces (a male-biasing effect).

Several limitations should be noted, however. First, training sets were of relatively small size (40–120 faces), limiting the leeway for training more accurate models. Second, the ratings used for human validation were obtained from neutral poses (control study, Supplementary Material) and not from the actual faces used in Experiment 3, and there were several missing values. Thus, they do not capture all the variations between stimuli used in Experiment 3. While a larger set of faces could have been manufactured for use in Experiment 3, along with obtaining their gender typicality ratings, it was considered preferable to use the very same set of faces in Experiments 1–2. Indeed, it allowed a direct comparison between human and machine categorization accuracy. Finally, our analysis relied on correlations that certainly do not imply causation: for example, one could imagine that machine classification log-odds from feature-based models correlated with mean human classification accuracy not because humans actually relied on these features, but because those features are precisely tracking another component of interest in human perception—for example, perceived anger intensity. A more definitive conclusion would require a manipulation of featural cues (and second-order relations) as is usually done in studies with artificial faces (e.g., Oosterhof and Todorov, 2009). Here, we chose to use real faces: although they permit a more hypothesis-free investigation of facial representations, they do not allow for fine manipulations.

That a feature-based model successfully replicated the human pattern of errors does not imply that such errors were entirely stimulus driven. Indeed, a feature-based strategy may or may not be hypothesis-free: for example, it may directly reflect stereotypical or experiential beliefs about gender differences in facial features (e.g., that males have thicker eyebrows) so that participants would use their beliefs about what males and females look like to do the task—beliefs that are reinforced by cultural practices (e.g., eyebrow plucking in females). In fact, a feature-based strategy could be entirely explicit (Frith and Frith, 2008); anecdotally, one of the youngest child participants explicitly stated to his appointed research assistant that “the task was easy, because you just had to look at the eyebrows.” On a similar note, it would be inappropriate to conclude that angry faces “objectively” resemble male faces as representations from Principal Component Analysis may be considered more objective than feature-based representations. Rather, it is the case that a specific, feature-based representation of angry faces resembles that of male faces. This point applies to other experiments in which a conjoinment of variant or invariant facial dimensions was explored computationally using human-defined features (e.g., Zebrowitz and Fellous, 2003; Zebrowitz et al., 2007, 2010). It appears then that the choice of a particular representation has profound consequences when assessing the conjoinment of facial dimensions. Restricting oneself to one particular representation of faces or facial dimensions with the goal of emulating an “objective” perception may not be realizable. Evaluating multiple potential representational models may thus be the more advisable strategy.

## General discussion

Overall, the results established the biasing effect of anger toward male gender categorization using signal detection analyses. The effect was present in adults as well as in children as young as 5–6 years of age, and was also evident with other-race faces for which anger had no effect on perceptual sensitivity.

The present results (1) are in accord with those of Becker et al. (2007) who reported that adults categorized the gender of artificial male vs. female faces more rapidly if they were angry, and female vs. male faces if they were smiling, and (2) replicate those of Hess et al. (2009) who reported that adults took longer to categorize the gender of real angry vs. smiling Caucasian female faces, but observed no such effect in Caucasian male faces. Similarly, Becker et al. (2007) found that adults were faster in detecting angry expressions on male vs. female faces, and in detecting smiling expressions on female vs. male faces. Conversely, Hess et al. (2004) found that expressions of anger in androgynous faces were rated as more intense when the face had a female rather than male hairline, a counter-intuitive finding that was explained as manifesting a violation of expectancy. Here, we complement the prior findings taken together by providing evidence for a male-biasing effect of anger using signal detection analyses, real faces, and a relatively high number of different stimuli.

We did not observe an opposing facilitation of gender categorization of female smiling faces, as could be expected from the results of Becker et al. (2007) and Hess et al. (2009), probably because in the present study, facial contours were partially affected by cropping. Furthermore, our results differ from those of Le Gal and Bruce (2002) who reported no effect of expression (anger, surprise) on gender categorization in 24 young adults, a null finding that was replicated by Karnadewi and Lipp (2011). The difference may originate from differences in experimental procedure or data analysis; both prior studies used a Gardner paradigm with a relatively low number of individual Caucasian models (10 and 8, respectively) and analyzed reaction times only, while reporting very high levels of accuracy suggestive of a ceiling effect [in fact, 22 participants from Le Gal and Bruce (2002) that had less than 50% accuracy in some conditions were excluded; not doing so would have violated assumptions for the ANOVAs on correct reaction times].

The findings yield important new information regarding the development of the angry-male bias. In particular, the male-biasing effect of anger was fairly constant from 5 to 6 years of age to young adulthood; the extensive social observation gained during schooling does not seem to impact the bias. This result is in accord with recent reports by Banaji and colleagues (Dunham et al., 2013; Cogsdill et al., 2014) showing that even belief-based interactions in the categorization of faces appear in their adult form much earlier than expected and do not appear to require extensive social experience. For example, Caucasian children as young as 3 years of age (the youngest age studied) were as biased as adults in categorizing racially ambiguous angry faces as Black rather than Caucasian (Dunham et al., 2013), an implicit association usually understood to reflect stereotyping (Hehman et al., 2014). Similarly, children aged from 3 to 5 stereotypically associated maleness with anger in cartoon faces (Birnbaum et al., 1980). Such biases may begin to develop in early infancy, a developmental period characterized by the emergence of gendered face representations rooted in visual experience (Quinn et al., 2002). Indeed, studies of racial prejudice have demonstrated a link between the other-race effect, a perceptual effect developing in infancy, and belief-based racial biases that are apparent from early childhood through adulthood such as associating other-race African faces with the angry expression (Xiao et al., 2015). It is possible that similar trajectories from perceptual to social representations may be found for gender. For example, a recent, unpublished study found that 3.5-month-old infants preferred a smiling to a neutral female expression, but preferred a neutral to a smiling male expression (Bayet et al., manuscript under review), suggesting an early association between female faces and positive emotions that results from differential perceptual or social experience with female caregivers. Such an early association could be a precursor to the increased performance of 5–6 year old children on smiling female faces that was observed in Experiment 2. Future studies on the developmental origins of stereotypes should focus on (1) finding precursors of stereotypes in infancy, and (2) bridging the gap between infancy and early childhood, thus providing a basis for early intervention that could curtail formation of socially harmful stereotypes.

Here, the male-biasing effect of anger appeared to be at least partially mediated by featural (e.g., brow thickness) and second-order (e.g., brow to eye distance) cues. While children have been reported to be less sensitive than adults to second-order relationships in some studies (e.g., Mondloch et al., 2002) and are less accurate in identifying facial emotional expressions (Chronaki et al., 2014), their encoding of featural information appears already mature at 6 years of age (Maurer et al., 2002) and they can recognize angry and smiling expressions most easily (Chronaki et al., 2014). Thus, the stability of the male-biasing effect of anger does not contradict current knowledge about children's face processing skills.

As discussed above, neither our behavioral nor our computational findings allowed us to embrace a particular mechanism for the male-biasing effect of anger, i.e., whether it was stimulus driven (an inherent conjoinment of dimensions) or stemmed from belief-based inferences. The findings are, however, relevant to the ongoing debate about the nature of face representations in the human brain. As stated by Marr (1982), any type of representation makes some kind of information evident while obscuring other kinds of information, so that studying the nature and origin of representational processes is at the heart of explaining low, middle, and high level vision. Various types of face representations have been proposed. For example, an important study in rhesus macaques found face-specific middle temporal neurons to be tuned to particular features or their combination while being affected by inversion (Freiwald et al., 2009). Other studies in humans have (1) emphasized the role of 2-D and 3-D second order relations in addition to features (Burton et al., 1993), and (2) argued for a double dissociation of featural and configural encoding (Renzi et al., 2013). An opposing line of argument has been advanced for a role of unsupervised representation analogs to Principal Component Analysis (Calder and Young, 2005) or Principal Component Analysis combined with multi-dimensional scaling (Gao and Wilson, 2013) or Gabor filters (Kaminski et al., 2011). All of those potential representations are fully compatible with the general idea of a face space (Valentine, 2001) since the face space may, in theory, present with any particular set of dimensions. Here, we provide additional evidence supporting the importance of features and second-order relations in the human processing of faces, and argue for the need to systematically consider various representational models of face processing when determining whether performance is stimulus driven, and to evaluate their respective contributions in perception depending on task, species, and developmental stage.

In conclusion, the present results indicate that the angry-male bias, whether stimulus- or belief- driven, does not require extensive social interaction with school-age peers to develop. It is in evidence as early as 5 years of age, and appears remarkably unaffected by experience during the primary grade levels, a developmental period that presumably includes observation of males engaging in aggressive acts.

## Author contributions

Study design was performed by LB, KL, OP, PQ, and JT. Data acquisition was conducted by LB, OP, and JT. Data analysis was performed by LB. All authors contributed to data interpretation, approved the final version of the article, revised it critically for intellectual content, and agree to be accountable for all aspects of the work.

### Conflict of interest statement

The authors declare that the research was conducted in the absence of any commercial or financial relationships that could be construed as a potential conflict of interest.
