# Supplementary material for: Angry facial expressions bias gender categorization in children and adults: behavioral and computational evidence
Source: Front Psychol. 2015 Mar 26;6:346. doi: 10.3389/fpsyg.2015.00346 (PMC4374394; doi:10.3389/fpsyg.2015.00346)
Supplement: Supplementary file 1 [file DataSheet1.PDF]

Supplementary Material

A. Control study

1. Material and methods

1.1. Participants and data preprocessing

Twenty four adult participants (mean age: 19.65 years, range: 16-24 years, 3 men) from a predominantly Caucasian environment participated in the control study. None had participated in Experiment 1. All gave informed consent and had normal or corrected to normal vision. The experiment was approved by the local ethics committee ("Comité d'éthique des centre d'investigation clinique de l'inter-région Rhône-Alpes-Auvergne", Institutional Review Board). One participant was excluded due to extremely long reaction times. Trials with a reaction time below 200 ms or above 2 standard deviations from each participant's mean were excluded, resulting in the exclusion of 4.68% data points.

1.2. Stimuli

One hundred four face stimuli of unique identities were selected from the Karolinska Directed Emotional Face database (Lundqvist et al., 1998), the NimStim database (Tottenham et al., 2002, 2009) and the Chinese Affective Picture System (Lu et al., 2005) database under their neutral frontal view versions. Faces were of different races (Caucasian, Chinese) and genders (female, male). The 104 faces (60 Caucasian, 44 Chinese) had the same identities as the faces used in Experiments 1-3, but were in neutral expression. A remaining 16 of the 120 faces used in Experiments 1-3 had no neutral expression available in the databases. Luminance, contrast, and eye position were matched as in Experiments 1-3. See **Figure 1B** for examples of the stimuli used.

1.3. Procedure

The general procedure was similar to that of Experiment 1, but the participants had to use the mouse to rate the gender typicality of each face on a scale underneath the face going from 0 (not very masculine or feminine) to 10 (very masculine or feminine). The face and scale remained on the screen until the participant responded. Each participant's rating and response time were recorded.

Each session began with 4 training trials that were identical to the experimental trials except that the faces were 2 females and 2 males randomly selected from the same set of 26 training faces used in Experiments 1-2. Each trial terminated with feedback to the participant showing which rating had been selected. Participants then performed 8 blocks of experimental trials. Half of the blocks included Caucasian faces (15 trials per block) and the other half included Chinese faces (11 trials per block). Blocks and trials were ordered as in Experiments 1-2.

1.4. Data analysis

Analyses were conducted in Matlab 7.9.0529. Data and code are available online at http://dx.doi.org/10.6084/m9.figshare.1320891

2. Results and discussion

Mean ratings for each stimulus category are presented in **Supplementary Table 1** along with the emotional properties of the stimuli used in Experiments 1-3. Overall, female faces were judged to be less gender typical than male faces. The mean ratings of each face were used as a control covariate in Experiments 1-2, and provided a human validation for the models in Experiment 3.

The gender typicality ratings of neutral poses obtained in this experiment effectively control for differences in perceived gender typicality for the stimuli used in Experiment 1-3. The effects of each stimulus category (Race, Gender, Emotion) in Experiments 1-2 cannot be attributed to the perceived gender typicality of the models. It should be noted, still, that since the ratings were obtained predominantly from young female adults they may not accurately capture the gender typicality of the faces as perceived by children or by the general population.

B. Supplementary Tables and Figures

Supplementary Figure 1. Gender categorization accuracy in Experiments 1 (adults) and 2 (children). Each star represents a significant difference between angry and smiling faces (paired Student t-tests, $p < 0.05$, uncorrected).

		Chinese faces				Caucasian faces			
		n	Hit rate (%)	Intensity (1-9)	Rating (0-10)	n	Hit Rate (%)	Intensity (1-9)	Rating (0-10)
Anger	male	10	88.4 ± 5.4	6.6 ± 0.5	5.8 ± 1.4	10	93.1 ± 9.5	6.6 ± 0.8	6.3 ± 1.4
	female	10	87.4 ± 7.8	6.5 ± 0.6	4.0 ± 1.3	10	92.3 ± 8.4	6.2 ± 0.6	4.8 ± 1.5
Smiling	male	10	99.4 ± 0.9	6.8 ± 0.4	6.9 ± 0.7	10	99.4 ± 0.8	6.8 ± 0.4	6.2 ± 1.4
	female	10	99.1 ± 0.6	6.8 ± 0.4	5.0 ± 1.7	10	98.8 ± 1.6	6.8 ± 0.3	5.4 ± 1.6
Neutral	male	10	88.7 ± 5.1	5.5 ± 0.2	6.3 ± 1.2	10	88.0 ± 3.8	5.3 ± 0.6	5.9 ± 1.0
	female	10	89.2 ± 6.9	5.6 ± 0.1	3.2 ± 1.5	10	88.9 ± 4.6	5.3 ± 0.2	5.2 ± 1.3
Overall		60	92,0 ± 7.2	6,3 ± 0,7	5.1 ± 1.9	60	93,4 ± 7.1	6,2 ± 0,8	5.6 ± 1.4

Supplementary Table 1. Mean emotional expression's hit rate, emotional expression's intensity, and gender typicality ratings of neutral poses for the stimuli used in Experiments 1-3. Hit rates and intensity ratings were obtained from the CFAPS documentation (unpublished data), the NimStim documentation (Tottenham et al., 2009) and a KDEF validation study (Calvo and Lundqvist, 2008). Emotion hit rates and intensities were used to match the stimuli across races and genders. Gender typicality ratings were obtained in a control study (**Supplementary Material**) and used a control covariate in Experiments 1-2 and as a validation tool in Experiment 3

Fixed effects	d.f.	χ^2	*p*

(Intercept)	1	33.56	<0.001
Race *	1	11.85	0.001
Gender *	1	3.95	0.020
Emotion	2	0.41	0.390
Mean gender typicality rating *	1	94.63	<0.001
Gender-by-Emotion *	2	30.55	<0.001
Race-by-Emotion *	2	29.86	<0.001

Supplementary Table 2: Best binomial GLMM of adult gender categorization accuracy. The model also included a random intercept and slope for participants. Significant effects are marked by an asterisk.

Fixed effects	d.f.	χ^2	*p*
(Intercept)	1	37.9	<0.001
Race *	1	21.05	<0.001
Gender *	1	6.34	0.010
Emotion	2	2.81	0.250
Age *	3	14.87	0.002
Mean gender typicality rating *	1	160.35	<0.001
Race-by-Gender	1	0.72	0.390
Gender-by-Emotion *	2	88.14	<0.001
Race-by-Emotion *	2	44.17	<0.001
Age-by-Race *	3	8.60	0.040
Age-by-Gender *	3	8.85	0.030
Age-by-Emotion	6	8.45	0.210
Race-by-Gender-by-Emotion *	2	10.22	0.006
Age-by-Gender-by-Emotion *	6	14.26	0.030

Supplementary Table 3: Best binomial GLMM of children's gender categorization accuracy. The model also included a random intercept and slope for the participants. Significant effects are marked by an asterisk.

Representation	Partition	Model	Correlation with human accuracy	
			r	*p*
Principal Component Analysis (PCA)	"familiar"	A	0.28	0.077
	"full set"	B	0.11	0.256
	"test angry"	C	0.04	0.704
Hand-Engineered features (HE)	"familiar"	J	0.39	0.013
	"full set"	K	0.25	0.007
	"test angry"	L	0.16	0.158

Supplementary Table 4: Correlation of human (adults and children) gender categorization accuracy and the absolute log-odds obtained at training by selected computational models of gender categorization. Correlations are Spearman correlation coefficients between absolute log-odds obtained by the model at training and mean human (children and adults) accuracy on the same faces. Log-odds from models that used principal components (PCA, models A-C) correlated less with

human accuracy than those from models that used hand-engineered features (HE, models J-L).
