# Supplementary figures and images for: Angry facial expressions bias gender categorization in children and adults: behavioral and computational evidence
Source: Front Psychol. 2015 Mar 26;6:346. doi: 10.3389/fpsyg.2015.00346 (PMC4374394; doi:10.3389/fpsyg.2015.00346)

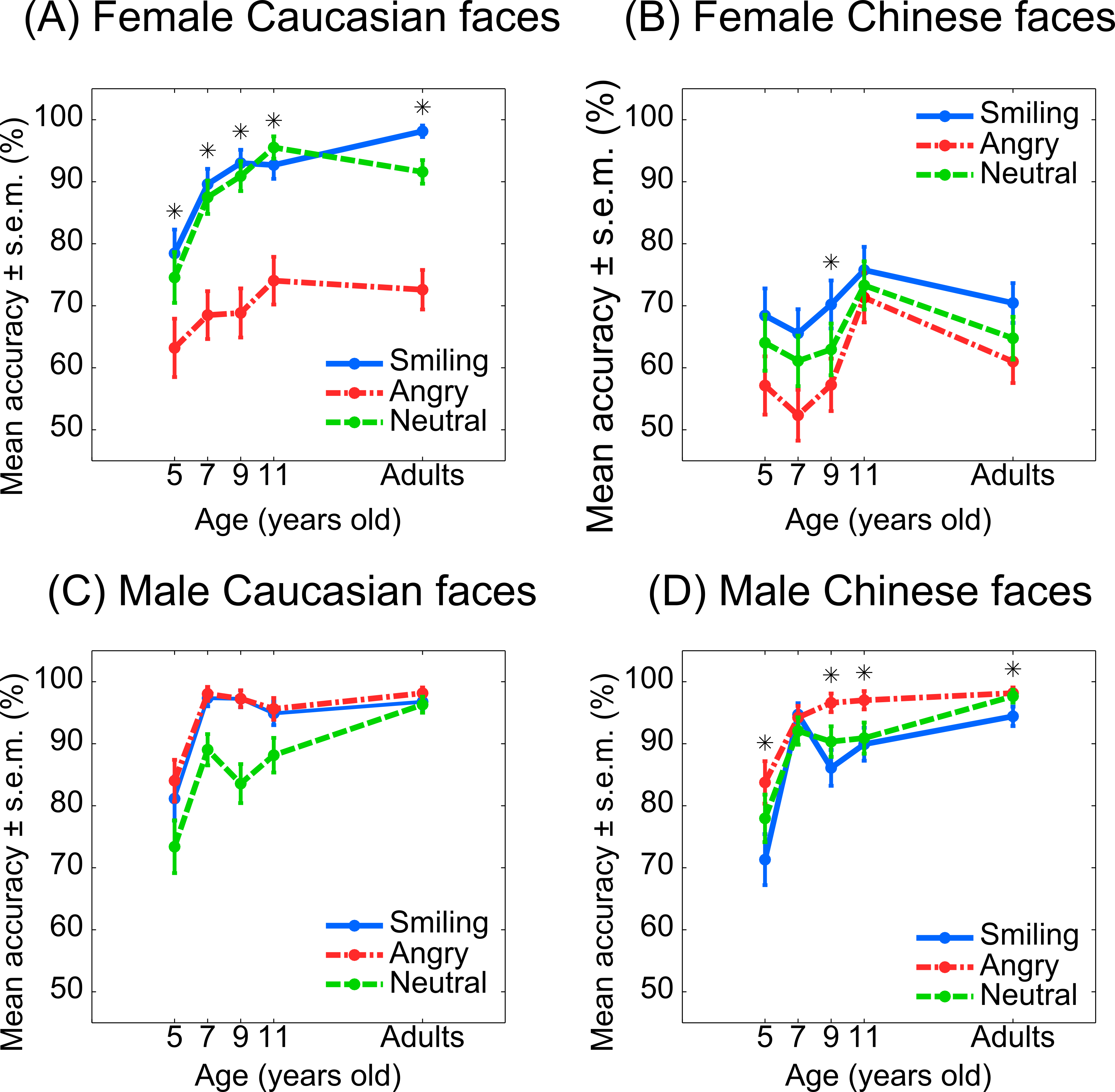

Supplement: Supplementary file 2 [file Image1.TIFF]
